# Your Automated Implantable Cardioverter Defibrillator Is Not a Bulletproof Vest but It Might Save Your Life

**DOI:** 10.5811/cpcem.2019.4.42086

**Published:** 2019-07-01

**Authors:** Tzlil Perahia, David S. Kleinman, Wassim G. Habre

**Affiliations:** * Crozer Chester Medical Center, Department of Emergency Medicine, Upland, Pennsylvania; † Crozer Chester Medical Center, Department of Cardiology, Upland, Pennsylvania; ‡ Crozer Chester Medical Center, Department of Acute Care Surgery, Upland, Pennsylvania

## Abstract

A 43-year-old male was brought to the emergency department as the highest level trauma activation with complaints of chest and arm pain after sustaining gunshot wounds (GSW). Initial workup was notable for superficial GSWs to the left chest and upper extremity with direct impact to the patient’s automated implantable cardioverter defibrillator. The patient underwent replacement of the device without rewiring and was discharged home without complications.

## INTRODUCTION

Automated implantable cardioverter defibrillator (AICD) placement is indicated in a variety of cardiac disease processes ranging from non-ischemic cardiomyopathy with reduced ejection fraction (EF) to recurrent ventricular arrhythmias, with a primary objective of reducing the risk of sudden cardiac death.^1^ A typical AICD is primarily composed of a header, containing the control circuitry, a power source, and the leads, which transmit an electrical impulse to the heart. The power source is commonly encased in a titanium alloy shell.^2^ On rare occasions an AICD has been reported to have altered a bullet’s trajectory and saved a patient’s life.^3^

## CASE REPORT

**Image 1:**
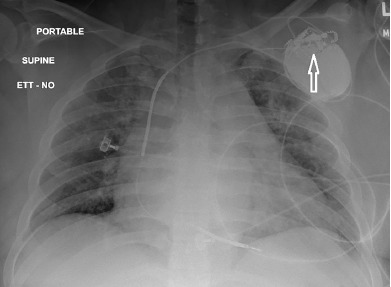
Anterior posterior chest radiograph showing cardiomegaly and damaged header of the automated implantable cardioverter defibrillator (arrow).

**Image 2:**
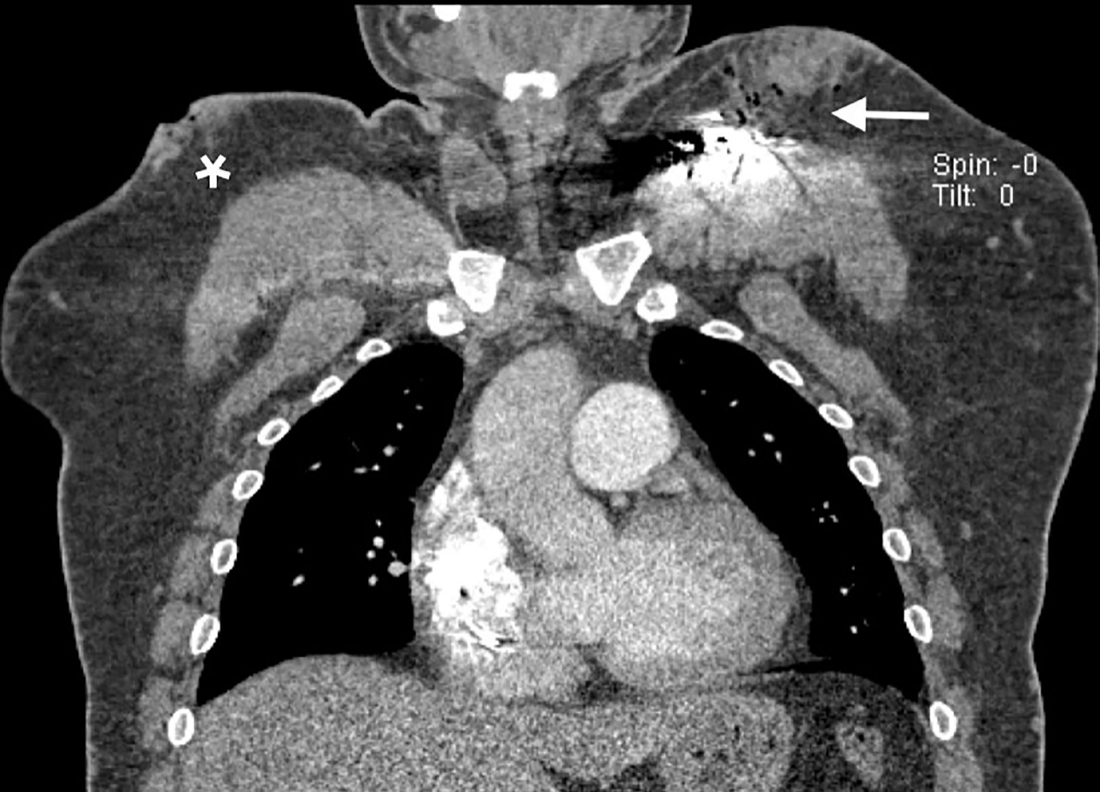
Representative computed tomography slice showing damaged automated implantable cardioverter defibrillator with surrounding subcutaneous air (arrow) as well as an additional gunshot wound to the right thorax (star).

A 43-year-old male was brought by emergency medical services to the emergency department (ED) as a prehospital trauma activation, having sustained multiple gunshot wounds (GSW) to the torso and right upper extremity. One of the GSWs was located overlying the patient’s AICD, which had been placed several years previously. The patient had additional GSWs, seemingly in a linear trajectory, across bilateral pectoralis muscles and right bicep. His past medical history was notable for dilated cardiomyopathy with EF less than 35%, paroxysmal atrial fibrillation, hypertension, diabetes mellitus, and history of pulmonary embolism currently on rivaroxaban. The trauma evaluation was negative for intrathoracic injury, and the patient was admitted for pain control and AICD interrogation. The initial chest radiograph demonstrated a grossly intact AICD. However, upon closer inspection the damaged circuitry of the header was visualized (Image 1). A representative slice of his chest computed tomography shows the damaged AICD with surrounding subcutaneous air, as well as an additional soft tissue deformity demonstrating an additional GSW (Image 2).

**CPC-EM Capsule**

What do we already know about this clinical entity?

There has been only one reported case of Automated Implantable cardioverter defibrillator (AICD) absorbing the impact of a gun shot wound (GSW) and preventing fatal injury.

What makes this presentation of disease reportable?

This case represents a rare complication of a common injury pathway, given increasing incidence of AICD implantations.

What is the major learning point?

Penetrating injuries to patients with AICD’s merit pacer interrogation during initial resuscitation to reduce risk of arrhythmia during periods of increased physiologic stress.

How might this improve emergency medicine practice?

This case raises awareness of a rare complication from GSW to the chest in patients with AICD and should hasten AICD interrogation in this select group of patients.

**Image 3:**
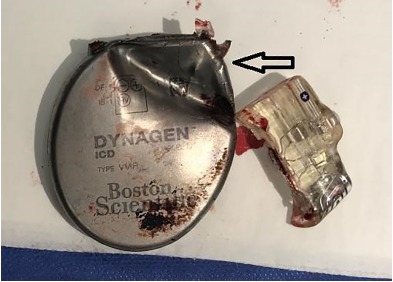
Explanted automated implantable cardioverter defibrillator showing damage to the pulse generator (arrow) and separation of the header.

The patient’s AICD was unable to be interrogated given the extent of the damage. During the operative exchange the header was found to be completely separated from the pulse generator, which had sustained direct damage from the bullet (Image 3). The leads remained intact. After an antibiotic rinse, the right ventricular coil and superior vena cava coil were reconnected and successfully tested with the new pulse generator. The patient recovered and was discharged to home with cardiology follow-up.

## DISCUSSION

From 2006–2012 over 300,000 patients underwent implantation of devices used to treat life-threatening cardiac conditions.^4^ The primary purpose of these devices is to administer an electrical impulse to the cardiac tissue with the goal of restoring a normal perfusing rhythm. These ICD devices have many modes of failure; however, the leads that conduct the electrical impulse are the most vulnerable component of the system.^5^ In the case described, the pulse generator and header suffered damage from the projectile impact while the leads remained unharmed. The failure of the AICD placed the patient at increased risk of developing a fatal arrythmia given the increased cardiac stress from his chest wall trauma and the patient’s innate risk from his dilated cardiomyopathy. Thankfully the patient did not decompensate or require any defibrillation/cardioversion during his hospitalization. His AICD was replaced without requiring rewiring of the leads, and he was discharged to home with cardiology follow-up.

## CONCLUSION

This case describes the second reported incidence of an AICD successfully deflecting a bullet in a patient presenting for multiple GSWs as well as successful pulse generator replacement without rewiring of the leads. Given the increasing implantation rates of cardioverter defibrillator devices, this case also demonstrates the importance of incorporating device interogation early on in patients sustaining penetrating chest wall trauma.
